# Creating a virtual leaf

**DOI:** 10.1093/aobpla/plad033

**Published:** 2023-06-04

**Authors:** Richard Harwood

**Affiliations:** School of Life and Environmental Sciences, University of Sydney, Camperdown, NSW 2006, Australia

**Keywords:** 3D modeling, leaf anatomy, photosynthesis, volume microscopy

## Abstract

When microscopy meets modelling the exciting concept of a ‘virtual leaf’ is born. The goal of a ‘virtual leaf’ is to capture complex physiology in a virtual environment, resulting in the capacity to run experiments computationally. One example of a ‘virtual leaf’ application is capturing 3D anatomy from volume microscopy data and estimating where water evaporates in the leaf and the proportions of apoplastic, symplastic and gas phase water transport. The same 3D anatomy could then be used to improve established 3D reaction-diffusion models, providing a better understanding of the transport of CO_2_ across the stomata, through the airspace and across the mesophyll cell wall. This viewpoint discusses recent progress that has been made in transitioning from a bulk leaf approach to a 3D understanding of leaf physiology, in particular, the movement of CO_2_ and H_2_O within the leaf.

## Teaser

Creating a 3D ‘virtual leaf’ from microscopy datasets allows for greater insight into the transport of carbon and water within leaves over multiple scales. A ‘virtual leaf’ blends volume microscopy, modelling and experimental data to better understand leaf anatomy and function. As the ‘virtual leaf’ becomes a refined tool plant physiologists will be able to run experiments computationally increasing our understanding of processes such as photosynthesis, respiration and water transport.

## Introduction

Microscopy has played a crucial role in our understanding of how plants function. It is incredible to think that over a century the idea of a novel observation has gone from a sketch of a new species to leveraging accelerated electrons to witness the internal anatomy of a plant at a resolution previously inconceivable.

Recent work has highlighted the importance of looking at anatomy in 3D (Brodersen and Roddy 2016; Lehmeier *et al.* 2017; Théroux-Rancourt *et al.* 2017, 2021; Earles *et al.* 2018; Earles *et al.* 2019; Harwood *et al.* 2020; Lundgren and Fleming 2020; Harwood *et al.* 2021; Borsuk *et al.* 2022; Momayyezi *et al.* 2022). Switching from 2D to 3D anatomy highlights that aspects of CO_2_ and water transport through leaves remain unresolved in established 1D and 2D approaches. Fundamental leaf processes, such as photosynthesis, respiration and transpiration occur across spatial and temporal scales and involve multiple tissue types throughout the leaf. Bulk leaf approaches fail to describe the influence that the geometry and biochemistry of different tissue types have on carbon and water exchange (Earles *et al.* 2019). Looking at leaf anatomy in 3D also highlights that the few 3D models that do exist are run on idealized interpretations of anatomy (e.g. Tholen *et al.* 2012; Xiao and Zhu 2017) or a blend of authentic and idealized geometry (Retta *et al.* 2023).

Investigating mesophyll conductance (the passage of CO_2_ from the sub-stomatal cavity the chloroplast stroma; *g*_m_) in 3D highlights how the size, shape and position of cells and organelles throughout the leaf influence *g*_m_ (Xiao and Zhu 2017)_._ Established methods for estimating *g*_m_ use a bulk leaf approach, meaning intercellular airspace (IAS) and chloroplast CO_2_ concentration are uniform across the leaf. In a real leaf with 3D anatomy accounted for, these CO_2_ concentrations would vary considerably depending on the 3D arrangement of cells and organelles along with their biophysical properties. One consequence of using a bulk leaf *g*_m_ is that responses to environmental conditions do not account for shifts in fine scale transport properties. For example, bulk leaf *g*_m_ is often observed to respond to irradiance (Douthe *et al.* 2011) but the processes behind the change are not incorporated in the interpretation. As irradiance changes and the effect on bulk leaf *g*_m_ is observed, several processes are potentially occurring, such as changes in the contributions of different mesophyll layers to CO_2_ fixation and production (Théroux-Rancourt and Gilbert 2017), and chloroplasts moving in response to light (Tsuboi and Wada 2011).

3D anatomy also has implications for understanding water transport. Incorporating authentic 3D leaf anatomy would increase our understanding of how hydraulic connections between cells and across the leaf are structured and hence how they operate. That is, authentic 3D leaf anatomy is essential in defining an accurate hydraulic design which in turn describes the phase, pathway and velocity of water movement from the xylem to the stomata. The challenge for hydraulic systems in the leaf lamina is supplying water to a large, thin and porous environment, a physiological task that has resulted in hydraulic and morphological diversity in leaves (Zwieniecki *et al.* 2007). Théroux-Rancourt *et al.* (2023) proposed a new way to look at the way airspace is connected to stomata. Their 3D leaf analysis breaks the IAS into discrete portions, that is, each stoma is associated with a specific IAS volume and was termed a stomata vapour shed. Their approach highlights that vapour movement throughout a leaf is not homogenous. The stomata vapour shed provides an excellent framework to think about a leave’s balancing act of stomata aperture, CO_2_ uptake and transpiration.

Currently, a small body of 3D anatomical data exists, even fewer with complementary gas-exchange measurements. Ideally, as 3D anatomical data become incorporated into plant physiology detailed information on internal leaf anatomy will be acquired for a few model species spanning diverse functional groups. 3D anatomical data could then be applied to a model of 3D carbon and water transport and the predictions could be compared to leaf-scale gas exchange, stable isotope measurements and other experimental data. This would begin to explain how different leaf’s structures affect photosynthesis and transpiration. If a 3D leaf model is adequately validated with sufficient experimental data, simulations (e.g. changing temperature, CO_2_ and irradiance) could be run solely on a 3D reaction-diffusion such as that developed in Tholen and Zhu (2011), but authentic anatomy from microscopy is used rather than simplified geometry to represent cells and organelles (Retta *et al.* 2023). In many ways the framework for such a model exists. For example, Evans *et al.* (2017) previously developed a model to describe the variation in absorption of light and photosynthetic capacity within different cell layers in the leaf, allowing interpretation of leaf-scale integrated chlorophyll fluorescence measurements when leaves were exposed to actinic light of differing wavelengths and intensities. Buckley (2015) and Buckley *et al.* (2017) presented 2D models that describe water transport throughout the leaf focusing on the site of evaporation and transport pathway. These models could be extended to 3D and be combined with models such as Tholen and Zhu’s (2011) 3D reaction-diffusion model of photosynthesis that accounts for CO_2_ production and fixation, and rates of CO_2_ diffusion through mesophyll cell walls, cytosol and plasma, mitochondrial and chloroplast membranes. If this approach were achieved, the term ‘virtual leaf’ is appropriate and represents methodological advancements that will increase our understanding of plant physiology.

It is essential that a ‘virtual leaf’ can account for temporal change, for example how the IAS and mesophyll cells change in response to drought. Momayyezi *et al.* (2022) investigated changes in 3D IAS under drought conditions. They coupled 3D imaging with experimental data to show that a leaf with higher porosity had less capacity to handle dehydration. This is the first-time leaf anatomy has been investigated on a temporal scale in 3D and represents a transition from a ‘snapshot in time’ to looking at 3D anatomy dynamically. Momayyezi *et al.* (2022) also took a suite of bulk leaf measurements such as stomatal conductance, photosynthesis and mesophyll conductance. To properly understand form-function relationships image acquisition would need to be at the same time scale as gas-exchange measurement (e.g. seconds) which is currently impossible. However, if anatomical change follows a trend over time, it should be possible to interpolate microscopy data to match physiological measurements. This combination of temporal anatomical and physiological data would allow for the movement of carbon and water inside a ‘virtual leaf’ to be tracked in real-time.

Here, I discuss the potential of the ‘virtual leaf’ to break through current knowledge gaps and highlight the challenges and opportunities that higher-dimension models bring to botany. A chickpea leaf imaged using serial block face scanning electron microscopy (SBF-SEM) at two different resolutions is presented to illustrate how even a snippet of a leaf has incredible structural diversity.

## Methods

### Growth conditions

Chickpeas (*Cicer arietinum* L.) were sown in 7-L pots. The pots were made up of bark-based potting mix and slow-release fertilizer (Osmocote, Scotts Australia Pty Ltd, Sydney, NSW, Australia) and were grown in a controlled environment room. The youngest fully expanded leaves were selected when plants were 6 weeks old and still in the vegetative stage. Leaf samples were collected mid-way along the leaves, avoiding the mid-vein.

### Sample preparation

The plant material was prepared using the protocol developed by Deerinck *et al.* (2010), with some modifications. 1 × 2 mm sections of wheat and chickpea were fixed in vacuum with 2.5 % glutaraldehyde, 3 % paraformaldehyde, 0.01% Triton X-100, 4 % sucrose, in 0.05 M phosphate-buffered saline pH 7.2 for 4 h then on a rotator for 15 h followed by washing in 0.05 M phosphate-buffered saline pH 7.2 three times for 5 min. Sample material was postfixed with 1.5% potassium ferrocyanide in 0.05 M phosphate-buffered saline with 2 % aqueous osmium tetroxide for 1 h then washed in milliQ H2O three times for 5 min. A freshly made solution of 1 % thiocarbohydrazide in milliQ H2O (placed in a 60 °C oven and agitated gently every 10 min for 1 h) was used to incubate the sample material for 20 min, the sample material was then washed in milliQ H2O three times for 5 min. The sample material was incubated in 2 % osmium tetroxide in milliQ H2O for 1 h, then washed in milliQ H2O three times for 5 min. The sample material was incubated for 15 h at 4 °C in 1 % uranyl acetate (aqueous) and then washed in milliQ H2O three times for 5 min. A fresh solution of en bloc Walton’s lead aspartate solution (0.7% of lead nitrate in l-aspartic acid, adjust to pH 5.5 with NaOH) was used to incubate the sample material for 30 min at 60 °C followed by the material being washed in milliQ H2O three times for 5 min. Sample material was then dehydrated with an ethanol series (15 %, 30 %, 50 %, 70 %, 80 %, 90 %, 95 %) for two 10-min periods at each concentration, then with 100 % ethanol for three 10-min periods. The sample material was placed in 25 % Procure 812 resin (Electron Microscopy Sciences, Hatfield, PA ) for 4 h, 50% for 15 h, 75 % for 4 h and 100 % for two 24-h periods, 100 % for 4 h in a vacuum and then 15 h and then embedded in 100 % for 72 h at 60 °C.

### Specimen mounting and SBF-SEM imaging

Once the resin had polymerized, samples were trimmed down to approximately 1 mm^3^, superglued onto a three-view specimen aluminium pin (Gatan, PEP6590) and allowed to cure overnight. The block face was trimmed at a zero-degree angle in an ultra-microtome (Leica Ultracut 7), and sputter-coated with a 10 nm layer of gold and silver painted on all exposed side edges to assist with block alignment and enhance conductivity.

Images were acquired using a variable pressure, field emission scanning electron microscope (Sigma VP; Carl Zeiss Microscopy, Jena, Germany) equipped with a Gatan 3View 2XP. Backscattered electron images were collected at a fixed working distance of 4.4 mm and standard 30 μm aperture. Imaging parameters for high-resolution chickpea: 4 kV, 16 Pa, 9000 × 8000 pixels, XY pixel size 6 nm and Z pixel size (slice thickness) 50 nm and pixel dwell time 1.5 μs. Pixel dwell time is the amount of time the electron beam spends scanning each pixel. Imaging parameters for low-resolution chickpea: 4 kV, 16 Pa, 15 000 × 9000 pixels, XY pixel size 15 nm and Z pixel size (slice thickness) 50 nm and pixel dwell time 1.5 μs. High-resolution images (XY pixel size 6 nm) were acquired to explore ultrastructure whilst the lower-resolution images (XY pixel size 15 nm) were acquired to explore whole cells.

### Segmentation and 3D analysis of SBF-SEM datasets

Image stacks were converted from DM4 to TIFF, in ImageJ. Many iterations were conducted to find a balance between TIFF size and clearly distinguishing ultrastructure, this resulted in a pixel being reduced from 6 × 6 × 50 nm to 20 × 20 × 50 nm for the high-resolution datasets (800 images in total) and 15 × 15 × 50 nm to 65 × 65 × 50 nm for the chickpea lower-resolution datasets (800 images in total). The reduced datasets were loaded and segmented individually in Avizo 9.2 (Thermo Fisher Scientific, Sydney, Australia). Segmentation was achieved by manually tracing a region of interest and using the interpolation feature within Avizo 9.2. The number of slices between manual segmentations was based on the geometry of the object.

## Results and Discussion

**Figure 1.**
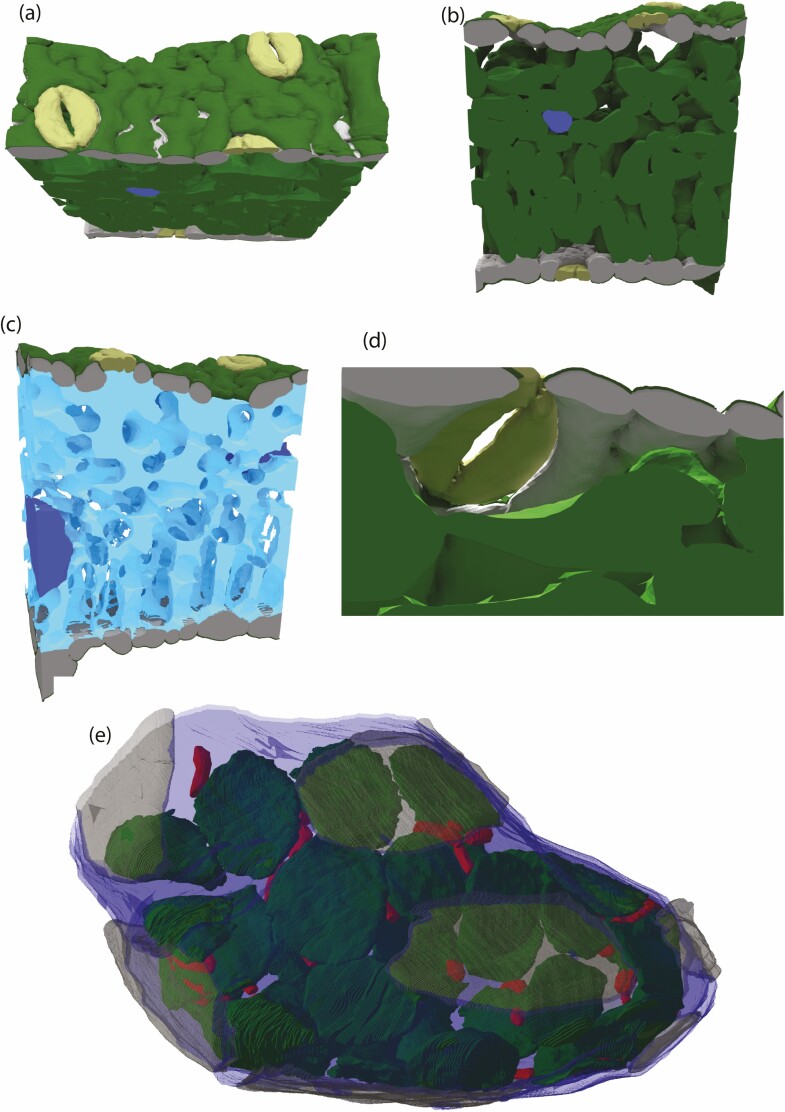
3D renders of a chickpea leaf looking at the adaxial (a), from the side (b), from the side showing the IAS (c), looking up at a sub-stomatal cavity (d) and a chickpea mesophyll cell (e). For a,b,c,d: stomata in yellow, cuticle in green, epidermal cells in grey, mesophyll cells in green, vascular bundle in blue, intercellular airspace (IAS) in light blue. For e: chloroplast in green, mitochondria in red, adjacent airspace in blue, adjacent cells in grey.

Qualitative results, in the form of renders, are used to conceptualize the framework for a ‘virtual leaf’. The goal here is that one could conceptualize a CO_2_ molecule making the journey from the atmosphere, via the stomata, through the IAS, the cell wall and finally the chloroplast envelope. Figure 1A shows the adaxial, Figure 1B shows a side on view and Figure 1C shows a side on view from an IAS perspective of a snippet of a chickpea leaf.

The abundance, size, position and aperture of stomata on a leaf’s surface directly influence the exchange of water and carbon with the atmosphere. Leaves with stomata on both the adaxial and abaxial surfaces have gas exchange advantages compared to those with stomata on one surface (Muir 2019). However advantageous this stomata design may be for enhanced gas exchange, it is rare, further demonstrating that increased leaf diffusional properties are never without trade-offs. For example, amphistomatous leaves have a greater susceptibility to pathogens (Drake *et al.* 2019). Lundgren *et al.* (2019) leveraged micro-CT, confocal imaging, and gas exchange on wild, domesticated, and transgenic lines of wheat and Arabidopsis. Their data gives a strong indication that mesophyll porosity is influenced by stomatal density and size. If we expand our investigation of the IAS beyond porosity (as exemplified by Earles *et al.* 2018) it becomes evident that two IAS structures can have the same porosity but differ immensely in their size, shape and connections (Borsuk *et al.* 2022). Ultimately the IAS is a complex 3D network for H_2_O and CO_2_ transport. Whilst metrics such as stomatal density are useful, a transition to the ‘virtual leaf’ approach would not only consider stomatal density, but the size and shape of the sub-stomatal cavity and its connections to the IAS (figure 1D) and the connections between the sub-stomatal cavity and the IAS (light blue, Figure 1C). Another reason to push toward the ‘virtual leaf’ is that leaf traits can be better considered together. For example, the stomata, sub-stomatal cavity and IAS influence overall leaf physiology (Théroux-Rancourt *et al.* 2023), but mesophyll cells cannot be so dense such that light cannot penetrate through the leaf. A bulk leaf approach does not provide sufficient information to thoroughly investigate the relationship between mesophyll cell packing and the IAS network.

Cell shape and IAS properties need to be considered together. For example, the lobe shape of rice mesophyll cells enhances the absorption of light and CO_2_ (Sage and Sage 2009). This demonstrates that at a constant cell volume invaginations and protrusions can increase cell surface area, subsequently increasing the surface area of mesophyll cells exposed to IAS (*S*_m_). It may be the case that increasing the cell surface area by increasing the complexity of shape allows for enhanced interception and absorption of light and CO_2_ while avoiding the cost of creating a new cell. An anatomical structure of high cell surface area and high porosity in the spongy mesophyll creates an environment that scatters a lot of light, which promotes absorption. Simonin and Roddy (2018) provide an excellent example of how different leaf anatomical designs influence physiology: the authors propose that smaller cells increase photosynthesis and that this switch in leaf architecture gave angiosperms a competitive edge. Cell size influences metabolism (Roddy *et al.* 2020) and *g*_m_ (Théroux-Rancourt *et al.* 2021) which supports the idea that increasing *S*_m_ via smaller and numerous mesophyll cells should result in higher photosynthesis. A ‘virtual leaf’ approach could help us better understand the fate of assimilated carbon, for example, if the increased assimilation is improving growth, reproduction, durability or defence (Bazzaz *et al.* 1987). Conceptually, a ‘virtual leaf’ could be coupled to a ‘virtual vascular system’ and ‘virtual roots’ allowing for assimilated carbon to be tracked across a ‘virtual plant’. From these interconnected models the volume and distribution of resources that make up a plant could be mapped in 3D making it possible to ascertain the fate of photosynthate. This understanding of the production and distribution of photosynthate along with the quantification of leaf construction cost could be leveraged to better understand the leaf economic spectrum (Wright *et al.* 2004).

Here is an inherently 3D example, this time focusing on water transport: An unresolved question that has been garnering attention of late is the transport of water from the xylem to the atmosphere. A leaf anatomy with cell and airspace properties that enhance photosynthesis (e.g. lots of small densely packed mesophyll cells) also needs to have an anatomical arrangement that can provide the necessary water throughout the leaf (Noblin *et al.* 2008; Brodribb *et al.* 2013) highlighting that optimizing CO_2_ assimilation cannot come at the cost of desiccation. For example, would an idealized cell density and airspace patterning that maximizes photosynthesis cope under water stress? Mesophyll cells are commonly conceptualized as spheres (Sun and Liu 2003), which, when packed together would theoretically have a contact point rather than a contact area. If a material has plasticity (e.g. a mesophyll cell) the contact area will be determined by the size of the spheres, the force at which this happens and the modulus of elasticity. Treado *et al.* (2022) developed a computer simulation where polygons (cells) change shape in response to stress and the way the cells interact with each other via attractive and repulsive forces. Simulations such as this provide temporal information on the formation and change of cell shape, IAS and *S*_m_ in the context of the leaf maintaining its capacity to be biomechanically sound. If a leaf is modified to increase *S*_m_ it could potentially limit the capacity to transport water, and while this may not be hugely consequential for a well-watered plant under water stress these cells could rapidly desiccate and there may be significant limitations to rehydration. A ‘virtual leaf’ approach to better understanding the role of anatomy regarding water stress would be to estimate the proportion of water transport pathways (apoplastic, symplastic and gas phase, Buckley 2015) and the sites of evaporation within leaves (Buckley *et al.* 2017) in 3D. This example is intended to highlight that we may initially look at cell shape and IAS to assess carbon sequestration capacity, then re-evaluate the same leaf architecture in regard to the pathway of water transport and the site of evaporation. A ‘virtual leaf’ approach could also see the Treado *et al.* (2022) simulation of cell growth coupled with the Buckley 2015 and Buckley *et al.* (2017) models of water transport. The merging of modelling approaches would increase our understanding of how different cell and airspace arrangements influence leaf function.

It is crucial to stress that what is shown here is a snippet of a leaf and ultimately the organelles (shown for one cell in Figure 1E) of each cell need to be considered. For example, just the snippet of chickpea leaf presented here would have hundreds of chloroplasts. In theory, each chloroplast would respond differently to external factors (e.g. light: Tsuboi and Wada 2011) and the response is influenced by cell shape. Therefore, an ideal virtual leaf would be able to trace light down the IAS and model individual chloroplast response. Retta *et al.* (2023) created a 3D reconstruction of a maize leaf using serial sections and a light microscope and modelled chloroplast movement based on the previous observations. This approach allowed the authors to compare different movement scenarios and better understand the relationship between photosynthesis rates, light intensities, CO_2_ concentrations and chloroplast movement. Furthermore, mitochondria also move in the cell and the amount of CO_2_ they respire at a given location would also influence photosynthesis and *g*_m_ (Cano *et al.* 2014), which due to imaging resolution could not be captured authentically by Retta *et al.* (2023).

It is important to keep a grounded perspective when attempting to capture in a laptop physiological process that evolved over millennia. As such, research efforts that contribute to the advancement of a ‘virtual leaf’ need to be assessed in terms of how they improve our limited understanding of plant physiology, not how they fail to capture complex reality. Xiao *et al.* (2022) exemplify an excellent approach to creating a ‘virtual leaf’. The authors use different microscopy modalities (micro-CT, confocal and transmission electron microscopy) to capture leaf anatomy at different spatial scales. This anatomical information is then pooled together and used to create a 3D structure that is representative of the microscopy. Whilst still somewhat ‘cartoon’ like in nature it is an excellent concession between 3D meshes that can be used in modelling software and authentic 3D anatomy. The authors validated their modelling assumptions with experimental data and termed the final product ‘eLeaf’.

Xiao *et al.* (2022) utilized ‘eLeaf’ to discover that changing leaf porosity had little influence on photosynthetic performance which aligns with the observations from Theroux-Rancourt *et al.* (2021) that cell size is much more important than porosity. It is worth noting that Theroux-Rancourt *et al.* (2021) took 3D anatomical data to estimate 2D CO_2_ diffusion and Xiao *et al.* (2022) took 2D anatomical data to estimate 3D diffusion. As 3D modelling approaches evolve, we will gain a better understanding of what level of detail is needed to answer specific research questions. Advancements in imaging techniques and artificial intelligence as a tool for segmentation (Théroux-Rancourt *et al.* 2020; Heinrich *et al.* 2021) will result in more refined and anatomically accurate 3D models. As these 3D models unravel the complexities of leaf physiology a ‘virtual leaf’ will emerge, representing a powerful tool that merges microscopy, modelling and experimental data.

## Data Availability

The SBF-SEM images and the corresponding masks are available from https://doi.org/10.5281/zenodo.7943403.

## Notes

**Form and Function. Chief Editor:** Kate McCulloh
